# Exploring Barriers and Facilitators to Dietary Assessment and Advice in the Paediatric Population Attending Dental Clinics: A Scoping Review

**DOI:** 10.1111/cdoe.13046

**Published:** 2025-05-27

**Authors:** Lauren Hallewell, Raul Bescos, Zoe Brookes, Robert Witton, Patricia Casas‐Agustench

**Affiliations:** ^1^ School of Health Professions Faculty of Health, University of Plymouth Plymouth UK; ^2^ Peninsula Dental School Faculty of Health, University of Plymouth Plymouth UK

**Keywords:** dentistry (MeSH), diet (MeSH), dietary advice, dietary assessment, paediatrics (MeSH)

## Abstract

**Objectives:**

This scoping review aimed to identify barriers and facilitators, from both dental clinic staff and caregivers, to effectively providing and implementing dietary assessment and advice (for both oral and/or systemic health) in dental clinics managing paediatric patients.

**Methods:**

A protocol was developed a priori (Open Science Framework‐ https://osf.io/bp4ts.) and followed the PRISMA‐ScR guidelines. Studies published in English from 1990 to December 2024 in MEDLINE, Cochrane Library, Embase and CINAHL databases were searched. Additional journal searches targeted articles on dietary assessment or advice in dental clinics treating paediatric patients (aged ≤ 18 years), exploring barriers and facilitators for caregivers and dental clinic staff.

**Results:**

Of 4736 studies identified, 32 were included, with 5 additional studies included from manual searching. Sixteen studies were quantitative, 13 were qualitative, and 8 mixed methods. Across studies, 77 barriers and 45 facilitators were identified in providing and implementing dietary assessment and advice in the paediatric population attending dental clinics. Results were mapped to the Theoretical Domains Framework. Common barriers for dental staff included time constraints and financial compensation, while caregivers cited controlling children's dietary habits as a major barrier.

**Conclusions:**

Understanding the main barriers and facilitators in providing and implementing dietary assessment and advice in dental clinics treating paediatric patients is crucial to improving preventive healthcare.

## 1 Introduction

Early childhood caries (ECC), a severe form of tooth decay, affects 514 million children globally, mainly due to poor diet and inadequate oral hygiene [1]. In the United Kingdom (UK), 23.7% of 5‐year‐old children experience tooth decay, and tooth extractions under general anaesthesia are the leading cause of hospital admission for children aged 5–9 in England [2, 3]. Among older children, dietary behaviours and oral hygiene practices continue to influence oral disease risk, with sugar‐sweetened beverage consumption and inadequate oral hygiene common contributors [4]. Adolescents (13–18 years) face additional challenges, including increased autonomy over food choices and susceptibility to peer and environmental influences, exacerbating oral health risks [5]. Untreated oral diseases can impose significant healthcare costs and affect children's education, quality of life and social skills [6, 7].

Oral diseases cause pain and discomfort, leading children and adolescents to avoid some hard foods, like fruits and vegetables, which are essential for health [7, 8]. Consequently, they may opt for processed foods that lack essential nutrients, exacerbating deficiencies that further compromise oral health [9]. Poor oral health, particularly periodontitis, is associated with increased risk of developing systemic diseases such as cardiovascular disease later in life, with diet also influencing the aetiology of both conditions [10]. Dietary advice addressing oral health is integral to both oral and systemic disease prevention [11].

Delivering dietary advice is a key preventive role for dental clinic staff, as emphasised by national guidelines. In the UK, the National Institute for Health and Care Excellence (NICE) and the prevention toolkit, Delivering Better Oral Health, stress delivering dietary advice to support oral health during routine dental examinations [11, 12]. Similarly, the American Dental Association policy statement recognises the critical link between nutrition and oral health, advocating for dietary guidance to promote healthy behaviours [13]. However, dietary advice in dental settings typically focuses on reducing sugar consumption and frequency and promoting fluoride and toothbrushing, rather than addressing broader and individual nutritional and health concerns [11, 14]. Usually, it does not cover nutrition and general health, which may indirectly affect oral health, although some dental clinics provide dietary advice regarding paediatric weight management, following body weight screenings [15, 16, 17]. Nevertheless, the quality of dietary advice in dental clinics can vary, is often brief and sometimes involves minimal patient interaction [18, 19, 20]. A 2014 scoping review [14] analysed the dietary advice provided by dental professionals and showed that factors like socio‐economic status, knowledge, cooking facilities/skills and motivation to change, which influence the success of dietary behaviour changes, are rarely considered. Lieffers et al. (2021) [21] examined nutrition care practices and barriers to dietary interventions by oral health professionals and dietitians in adults and children, focusing on dentition and periodontium. Only one review addressed barriers/facilitators to caries prevention in children [22] encompassing fluoride application, oral hygiene and dietary advice.

Thus, this scoping review aimed to identify the main barriers and facilitators dental clinic staff and caregivers' face when providing and implementing dietary assessment and advice for the paediatric population attending dental clinics. The term ‘dental clinic staff’ acknowledges the importance of interprofessional collaboration, involving dentists, dental nurses, dental therapists, dental hygienists and dietitians/nutritionists (in some American clinics) for dietary assessment or advice [23, 24]. These interactions are shaped by factors at all levels of public health: Upstream (policies and systemic factors), midstream (community and social influences) and downstream (individual behaviours and skills) [25]. This highlights opportunities for improving dietary advice and assessment for systemic and individual challenges. Additionally, this study aimed to investigate the types of dietary assessment and advice provided, focusing on studies that identify these barriers and facilitators.

## 2 Methods

This scoping review adhered to the PRISMA‐ScR (Preferred Reporting Items for Systematic Reviews and Meta‐analyses extension for Scoping Review) [26] and Joanna Briggs Institute [27] guidelines and was registered on the Open Science Framework https://osf.io/bp4ts.

### 2.1 Search Strategy

Search terms were derived from initial articles and tested across MEDLINE (EBSCO), Cochrane Database of Systematic Reviews (Wiley), Embase (Ovid) and CINAHL (EBSCO). Detailed search strategies are provided in Table S1. MEDLINE and CINAHL utilised MeSH terms, while Embase employed Emtree terms (including some exploded). The search spanned from 1990 to December 2024 and included only English‐language papers. Studies were selected to ensure comprehensive coverage of the literature, building on previous reviews that had identified papers discussing dietary assessment and advice [14] as these began to gain recognition in dental care settings [28]. Additional manual searches included nine journals such as *Journal of Paediatric Dentistry, Community Dentistry and Oral Epidemiology, British Dental Journal* and others, which can be found as Data S1, to account for variations in abstracting service depth and indexing [29].

### 2.2 Study Selection of Evidence

Papers from database searches were managed using EndNote X8.2 (Clarivate Analytics, PA, USA). Included studies, of any design (quantitative, qualitative or mixed methods), focused on barriers and facilitators to dietary advice and assessment in dental clinics for improving paediatric health. Guidelines, editorials, pre‐prints, abstracts, protocols and reviews (systematic, meta‐analysis, literature, scoping) were excluded to avoid duplication.

Studies involving participants aged 0–18 years were included. This age range was selected to capture the continuum of dietary behaviours and oral health across infancy, childhood and adolescence, recognising that these developmental stages present unique dietary and oral challenges and intervention needs [4]. In this review, ‘dietary advice’ encompassed dietary or nutritional information provided through written materials, brief discussions or dietary counselling aiming to improve paediatric overall and/or oral health. ‘Dietary assessment’ broadly referred to identifying dietary intake and habits, including methods like diet histories, 24‐h dietary recalls and verbal questioning. All dietary advice and assessments discussed were provided at dental clinics. In this study, ‘dental clinics providing paediatric care’ referred to locations where patients aged ≤ 18 years old receive dental treatment or advice related to oral health or oral care. Consequently, all paediatric studies including dietary advice and/or assessment provided by dental clinic staff were included in this review encompassing, triggers, reasons, views, experiences or beliefs. Supportive factors were classified as facilitators, while hindering factors were categorised as barriers. In quantitative studies, barriers and facilitators were defined as such if at least 10% of participants reported them, aligning with Lienhart et al.'s [22] systematic review threshold for significant population representation. For qualitative studies, data were extracted from direct participant quotations, following the methodology outlined by Lienhart et al. [22], and analysed with research team consensus.

### 2.3 Assessment of the Reporting Quality of Methodology

Articles were included regardless of quality to encompass the full scope of available literature.

### 2.4 Data Charting Process

Two reviewers (LH and PC‐A) independently screened all titles and abstracts for eligibility using Ryann (Ryann Systems Inc), a web‐based software designed to facilitate collaborative screening and decision making in scoping reviews, to identify studies that met the predefined inclusion criteria. Following this, full‐text papers were reviewed independently by the two reviewers and discrepancies were resolved with a third reviewer (RB).

Extracted and tabulated data included author, reference, geographical location, study aims, study design, healthcare professional delivering the dietary assessment/advice, type of dental setting (public, private, hospital, community, etc.) and provision of dietary assessment/advice.

Direct quotations and figures were coded to the 12 domains of version 1 of the theoretical domains framework (TDF) as either a barrier or facilitator, which capture influences of cognition, emotions, social and environmental factors that impact one's behaviour [30], using Nvivo 14 (QSR International). For direct quotations from qualitative studies, consensus with the third reviewer was necessary to determine inclusion in the data mapping process. To provide a comprehensive presentation of the data, the results were organised into four separate tables. These tables categorised the data based on stakeholder group and whether the findings represented barriers or facilitators.

The frequency of barriers and facilitators was calculated by counting the number of studies that reported each individual barrier or facilitator within a TDF domain. For instance, under the environmental domain, if ‘time constraints’ was reported as a barrier in five separate studies, it was recorded as five occurrences. Similarly, if a single study identified two distinct barriers within the same domain (e.g., ‘time constraints and ‘lack of resources'), each were counted as separate occurrences to reflect the diversity of insights. However, within a single study, each barrier or facilitator was counted only once per domain, to avoid redundancy, even if the same barrier was mentioned multiple times throughout the study.

## 3 Results

**FIGURE 1:**
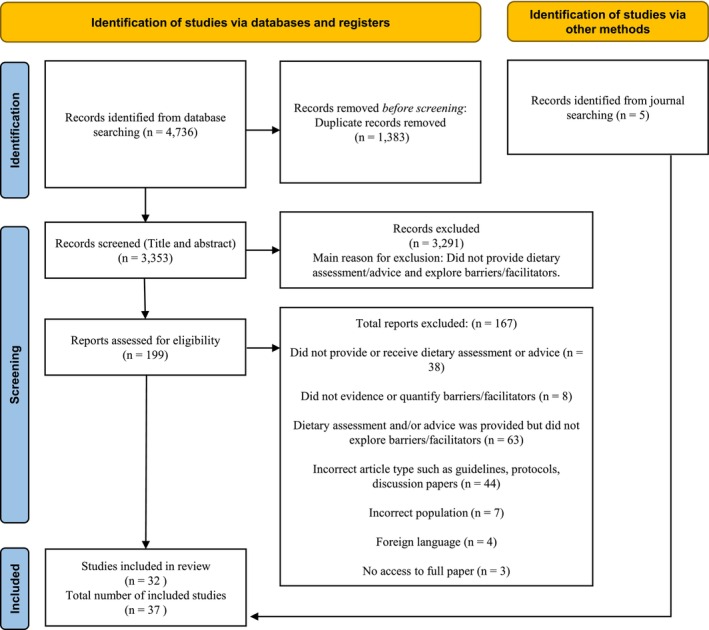
PRISMA flow diagram.

Database searches identified 4736 studies published between 1990 and December 2024. A total of 1383 duplicates were removed, resulting in 3353 studies screened for abstract and title (Figure 1). Of these, 199 were assessed for full‐text eligibility, with 32 included in content analysis. Five additional studies were added from manual database searches. Table S2 highlights the characteristics of each included study. The studies dated from 1994 to 2024 and originated from eight countries: the United States of America (USA) [31, 32, 33, 34, 35, 36, 37, 38, 39, 40, 41, 42, 43, 44, 45], UK [46, 47, 48, 49, 50, 51, 52, 53, 54, 55], Australia [56, 57, 58, 59, 60], India [61, 62], Japan [63], Jordan [64], Korea [65] and Trinidad [66]. Of these, seventeen were surveys/questionnaire [31, 33, 34, 35, 39, 40, 41, 42, 43, 45, 47, 48, 53, 55, 63, 64, 65], four were focus groups [44, 59, 66, 67], seven were interviews [32, 38, 46, 54, 56, 57, 60] and seven a combination of these methods [36, 37, 50, 51, 58, 61, 62]. One study conducted a content analysis of dental advice [52], while another was a retrospective study [49]. Nine studies involved caregivers [32, 36, 38, 44, 57, 60, 64, 66, 67], twenty‐two involved dental clinic staff [31, 33, 34, 35, 37, 39, 40, 41, 42, 43, 45, 47, 48, 51, 53, 54, 56, 58, 59, 61, 62, 65], and three assessed both caregivers' and dental clinic staffs' perspectives [46, 50, 63]. While the majority of included studies were exploratory in nature and did not involve direct dietary interventions, they were crucial in understanding downstream (individual‐level), midstream (organisational‐level) and some upstream (policy‐level) factors that could influence the success of dietary assessment and advice delivery and engagement in dental settings. These insights can serve as a foundation for designing future interventions and addressing challenges at all levels of public health.

The most commonly reported professions in the dental clinic were General Dental Practitioners, paediatric dentists, auxiliary staff such as dental nurses, dental hygienists, dental therapists, nurses and nutritionists/dietitians. A variety of barriers and facilitators were reported by dental clinic staff and caregivers to the provision and implementation of dietary assessment and/or advice. Tables 1, 2, 3, 4 present these findings mapped onto the TDF. Some TDF domains were not addressed in the literature, resulting in empty categories. The frequency of each reported domain can be found in Table S3.

**TABLE 1:** Dental clinic staff reported barriers mapped to each TDF domain.

Theoretical domain	Dental clinic staff reported barriers to providing dietary assessment and/or advice
Knowledge	Lack of knowledge in diet analysis [47], nutritional counselling [31, 45], initiating conversations on diet [45], discussions related to food insecurity [55] and health professional education [43]. Uncertainty about personal knowledge concerning obesity and dental caries [34]. Lack of familiarity with dietary guidelines [42, 56]. Clearer evidence linking obesity to oral disease could increase interest in advising patients about weight management [45]. Caregivers education [31, 54, 56], literacy levels [47] and ability to process information [63].
Skills	Lack of training in communication skills [45] and healthy eating counselling for weight management [34]. Lack of skills required for dietary counselling [34, 61, 62], dietary assessment [42, 62], including discussions related to food insecurity [55].
Social/professional role and identity	Concerns about legal risks [31, 45] and providing incorrect dietary information outside of one's scope of practice [56]. Lack of personal agreement with recommendations on breastfeeding practices [42]. Inconsistencies in dietary advice among general practitioners, doctors, nutritionists and dentists [50].
Beliefs about capabilities	Uncertainty about how to implement a nutritional programme [31]. Dietitians lack of confidence in providing information and counselling about oral health [35], as well as discussions related to food insecurity [55]. Perception about caregivers capabilities that they provide sweets to placate children [54].
Beliefs about consequences	Poor compliance of diet diaries from caregivers [47, 61, 62]. Caregivers lack acceptance of receiving dietary advice from a dental professional [34, 43, 45]. Practice staffs relationship with patients could be compromised when discussing nutrition [43, 45, 50]. Caregivers not following dietary instructions [33]. Dietary advice regarding obesity provided in the dental setting could take over from the main oral health message [50]. Dietary diaries are often missing information, compromising the validity of the assessment tool [49].
Motivation and goals	Lack of caregivers acceptance of nutritional advice from a dentist [45], willingness to complete a diet diary [46, 54], willingness to implement dietary advice [33, 54] or interest/motivation in diet [31, 35, 40, 42, 45]. Patient and caregiver resistance to accepting a referral to a dietitian in the dental setting [40]. Perception that dietary counselling does not change behaviours or it is challenging [34, 41, 42, 51, 56]. Personal beliefs regarding diet that unless sugar intake is under control then the other preventive efforts would be to little avail. It is a dentist's obligation to explain this to patients, and the rest is up to caregivers [51]. Providing dietary advice does not attract a ‘dollar figure’ [56]. Infant/toddler health is not a focus of practice [42].
Memory, attention and decisions	None identified.
Environmental context and resources	Time‐consuming nature of diet diaries [47, 61, 62] and providing dietary advice [31, 39, 40, 42, 43, 45, 54, 59], including discussions related to food insecurity [55]. Inadequate resources [39, 43, 45] and trained staff [31, 42, 45] for delivering nutritional advice. Lack of referral options to other supportive healthcare professionals [40, 45, 56]. Dietitians working in the dental clinic infrequently receiving patient referrals for nutrition counselling [35]. Cost of dietitians services in the dental clinic [35, 40]. Inadequate renumeration [45, 47, 54, 56, 61, 62] or reimbursement [31, 39, 40, 42, 45] to provide dietary assessment/advice, including discussions related to food insecurity [55] External environment making cariogenic foods cheaper than healthy foods [31, 43, 56], and marketing messages promoting cariogenic foods even from well‐intentioned nutrition programmes [37].
Social influences	Caregivers prioritising more pressing needs than children's sugar‐sweetened beverage consumption [58]. Patient socio‐economic level [31], including dental professionals providing unrealistic dietary advice for patients experiencing food insecurity [55]. Language or cultural barriers [39]. Family members have different child‐rearing principles (impacting child's eating problems) [63].
Emotion	Fear of offending patients and appearing judgemental toward caregivers and/or children [43, 45]. Fear that patients might feel upset or ashamed when discussing obesity (including dietary advice) [50]. Do not feel comfortable providing nutritional advice [39]. Providing obesity‐related services (including dietary advice) is challenging due to the emotional and personal nature of the topic [50].
Behavioural regulation	Existing beliefs that: People can eat a healthy diet if they choose to do so [34]. Obesity is caused due to a lack of discipline, bad parenting and is the persons fault, regardless of education levels [50]. Caregivers do not have control over the child's dietary behaviour [37]. Caregivers use excuses in order to ‘save face’ when not completing the diet diary [46].
Nature of the behaviours	Dietary behaviour is hard to change due to caregivers mindset [56].

**TABLE 2:** Caregivers reported barriers mapped to each TDF domain.

Theoretical domain	Caregivers' reported barriers to implementing and engaging with dietary assessment and/or advice
Knowledge	Lack of knowledge regarding bottle‐feeding practices [38, 60], hidden sugars in food products [44] and terminology used by dental professionals, including the term ‘tooth decay [60]. Conflicting advice on breastfeeding and caries provided by dentists versus doctors [66]. Unclear advice on the link between bottle‐feeding, oral health and systemic health [60].
Skills	None identified.
Social/professional role and identity	Caregivers can be unreceptive to oral health guidelines regarding bottle‐feeding suggestions [36, 38]. When dental professionals suggest ‘simple’ dietary changes, it can be an obstacle for a child with autism spectrum disorder [67]. Caregivers feel that dental professionals should recognise them as experts by virtue of their experience with their child's condition [67].
Beliefs about capabilities	Lack of control over children's dietary behaviours [44, 46, 66], including when they are outside supervision (i.e., going to the shops and buying sweets with friends) [46]. Difficulty in limiting child's sugar intake due to behavioural temperament [57], including instances where children do not respect caregiver authority [61]. Challenges as children get older and gain more freedom, allowing them to ask for sugary food products [60]. Difficulties in getting children to give up the bottle, as they might not understand and could become upset [36].
Beliefs about consequences	Fear of being shamed by the dental professional (when using a diet diary) [46]. Dentists disbelieving what the caregivers are reporting [44]. View that dentists are ‘marking’ diet histories as if it were a test [46, 62]. Childrens adverse behavioural consequences if denied sweets/chocolate [57]. Potential adverse consequences of providing repeated dietary messages (related to obesity), could lead to obsessive dietary behaviours in children [50].
Motivation and goals	Absence of belief of usefulness of diet diaries [62].
Memory, attention and decision processes	Caregivers did not recall diet advice [64]. Forgetting the food diary when attending the dental clinic [46].
Environmental context and resources	Shopping environment and food advertisement is negatively influencing children's dietary choices (related to oral health) [57, 60]. A paper dietary diary is labour intensive and sometimes inaccessible [46]. Leaflets provided in dental settings can provide confusing/conflicting dietary advice to national dietary guidelines (e.g., illustrated image of a large portion of cheese as a snack) [52].
Social influences	Influence of extended family members on their child's diet (related to oral health), in particular, grandparents [44, 57, 63, 66]. External influence of children's friends dietary behaviours [66]. Struggling to fit the diet diary into everyday life, in particular caregivers who are working full time and with children at the age of school attendance [46, 61].
Emotion	Fear of being judged by dental professionals [46, 57] and appearing careless [46]. Dentists using paternalistic language when talking through dietary diaries [46]. Feeling terrible and guilty for not having control over child's diet [57, 66]. Guilt for not breastfeeding for those who are unable to do so [60]. Children fear of being reprimanded so not disclosing everything they eat in the diet diary [46, 48].
Behavioural regulation	Altering diet history accounts to be met with approval [46]. Adopting defensive behaviours as a mechanism to appear favourable in dentists eyes (i.e., losing the diary) [46]. Caregivers personal perception about obesity aetiology: belief it is down to laziness [50].
Nature of the behaviours	Difficulty in breaking dietary habits that contribute to the cariogenic process (e.g., bottle‐feeding practices) [60].

### 3.1 Barriers

Mapping barriers onto the TDF revealed that 11 domains were influential for both dental professionals and caregivers (Table S2 and Tables 1). However, ‘Memory, attention and decision processes’ was absent for professionals, while ‘Skills’ was absent for caregivers. The most frequently reported barriers were in the domains of ‘Knowledge’, ‘Environmental context and resources’ and ‘Beliefs about consequences’.

Barriers related to ‘Knowledge’ included limited familiarity with dietary guidelines, lack of tracking in diet analysis and counselling, and uncertainty linking obesity to oral health among dental clinic staff, while caregivers faced barriers related to dietary advice for younger children (e.g., breastfeeding and bottle‐feeding practices), conflicting advice from health professionals and limited awareness of hidden sugars in foods.

Barriers related to ‘Environmental context and resources’ included time constraints, inadequate resources, insufficient staff training and limited reimbursement to provide dietary advice as barriers to providing dietary advice for dental professionals, while caregivers noted challenges such as the negative influence of shopping environments, labour‐intensive paper diet diaries and conflicting advice in educational materials.

Barriers related to ‘Beliefs about Consequences’ included dental professionals' concerns about caregiver compliance with diet diaries, acceptance of dietary advice and potential strain on relationships when discussing nutrition, while caregivers expressed fears of being judged by dental professionals and concerns about adverse behavioural consequences for children when sugary treats were restricted.

### 3.2 Facilitators

Facilitators, which were less commonly reported than barriers, highlighted notable gaps (Tables 3 and 4 for dental clinic staff and caregivers, respectively). For caregivers, facilitators were absent in five domains including ‘Skills, beliefs about capabilities’ and ‘Social influences’. Similarly, for dental professionals, four domains, such as ‘Beliefs about capabilities and emotion’ were unpopulated, suggesting that certain areas may not currently serve as facilitators in this context (Table 1).

**TABLE 3:** Dental clinic staff reported facilitators mapped to each TDF domain.

Theoretical domain	Dental clinic staff reported facilitators to providing dietary assessment and/or advice
Knowledge	Research or personal knowledge demonstrating a link between oral health, obesity and general health [34, 50, 56]. Feel they have adequate knowledge to provide dietary counselling [55].
Skills	Comfort [31, 39, 43] and confidence [31] in providing nutritional advice, including obesity prevention [43] and general/non‐caries associated nutritional counselling [31]. Using dietary assessment tools to then tailor dietary advice [48, 56]. Dental curricula should place additional emphasis on nutrition not associated with caries [31]. Interested in continuing education that includes topics on nutrition and healthy lifestyles [31, 56]. Interprofessional education with dietitians as part of dentistry studies [40]. Additional training for how to tackle difficult conversations around food security and how to provide appropriate dietary advice [55].
Social/professional role and identity	It is a professional responsibility to educate caregivers on nutrition and the sugar content of foods [37] and provide dietary counselling [55]. Providing advice on sugar‐sweetened beverages and obesity can enhance professional credibility with caregivers [45]. The dental profession has a responsibility to offer dietary and obesity‐related advice [43, 45, 56]. Fostering interprofessional collaboration with other healthcare professionals (e.g., paediatricians, dietitians) [35, 37, 40] and auxiliary dental personnel for dietary advice and assessment [40, 47, 54]. Belief that caregivers are receptive to receiving dietary information and nutritional counselling from a dietitian in the dental clinic [35]. A dental office is seen as an appropriate environment for a nutrition expert [31].
Beliefs about capabilities	None identified.
Beliefs about consequences	Belief that nutritional counselling is effective and can change behaviours [33, 34, 41]. Belief that caregivers are receptive (or somewhat) to dietary advice (including non‐caries associated advice such as obesity) [31, 45]. Discussing children's health in the view of overall health improvements to engage caregivers, instead of telling caregivers what to do [56]. All age groups could benefit from general nutritional counselling if these services were offered in a dental office [31]. If dental professionals were given the time to provide dietary advice, it could save the National Health Service money in the future [54].
Motivation and goals	Caregiver and child motivation facilitates the use of diet diaries [47]. Perception that social class influences caregiver motivation, with middle‐class caregivers being more motivated [54]. A personal belief that it is rewarding to talk about nutrition and a desire to counsel patients about nutrition [34]. Personal perception that nutrition advice is effective [39, 41, 65]. Nutritional counselling [41] and SSB education [45] is an important aspect of oral health care. Dental professionals use dietary diaries to provide either general diet advice, addressing a common aspect among various issues or picking a key issue that they perceive is the most important to then provide dietary advice [48]. Belief that diet counselling will benefit the dental practice [51].
Memory, attention and decision processes	The presence of dental caries can impact the frequency of providing nutritional advice [39]. Children without dental caries tended to be questioned less frequently about dietary intake in comparison to children experiencing dental caries [54]. Asking both child and caregiver to keep the diet diaries [47]. Using diet as a common risk factor to address both obesity and caries dietary advice [56]. Normalising discussions around food insecurity as part of assessments, to reduce the stigma [55].
Environmental context and resources	Using a dietary diary resource to assess dietary intake [56], monitor patients dietary behaviour [47, 56], and prompt behaviour change [47]. TV screens to provide information in the waiting room regarding obesity and oral health to deliver dietary messages [56].
Social influences	None identified.
Emotion	None identified.
Behavioural regulation	Providing reassurance to caregivers by emphasising they are not judging them [46].
Nature of the behaviours	None identified.

**TABLE 4:** Caregivers reported facilitators mapped to each TDF domain.

Theoretical domain	Caregivers' reported facilitators to implementing and engaging with dietary assessment and/or advice
Knowledge	Leaflets that provide clear, colourful and simple information to avoid information overload [60]. Dental professionals and WIC staff reinforcing the same dietary message [32].
Skills	None identified.
Social/professional role and identity	None identified.
Beliefs about capabilities	Feeling in control of the child's diet when they are young [60]. Dentists and doctors providing dietary advice that aligns with caregivers' advice to children, as children are more likely to listen to professionals [46].
Beliefs about consequences	None identified.
Motivation and goals	None identified.
Memory, attention and decision processes	None identified.
Environmental context and resources	A smartphone app would be beneficial to help record diet diary information [46]. Engaging displays in the dental waiting room, including advice regarding how much sugar is in food/drink stuffs [32, 44].
Social influences	Caregivers encouraging children to complete the diet diary themselves or working through the diet diary with their child to overcome the barrier of caregivers not having enough time [46].
Emotion	Appreciation toward non‐confrontational, patient‐centred, individualised advice [57, 59]. After the dental professional provides nutrition advice, it can feel like a huge privilege to ask the dental professional for specific personal dietary advice [32].
Behavioural regulation	Diet diaries can be a useful tool to support caregivers in identifying where dietary changes can be made [46].
Nature of the behaviours	Encouraging the child to get into good eating habits from a young age to prevent cavities [66].

Analysis of facilitators revealed notable differences in the most prominent TDF domains between dental clinic staff and caregivers. For dental clinic staff, the ‘Social/professional role’ domain emerged as the significant facilitator, emphasising a strong sense of professional responsibility to educate caregivers on nutrition. Facilitators in this domain also included fostering interprofessional collaboration with other healthcare professionals and auxiliary staff, as well as the perception that caregivers were receptive to nutritional counselling in the dental setting.

In contrast, facilitators for caregivers were most prominent in the ‘Emotion’ and ‘Environmental context and resources’ domains. Facilitators in the ‘Emotion’ domain included caregivers' appreciation for non‐confrontational, patient‐centred advice. Within the ‘Environmental context and resources’, caregivers valued the potential use of smartphone apps to assist with completing dietary assessments and engaging displays in dental waiting rooms that provided, practice advice, such as information on sugar content.

### 3.3 Dietary Advice

While many of the included studies did not describe formalised dietary interventions, they explored key barriers and facilitators to providing and implementing dietary assessment and advice in dental clinics. Where mentioned, the types of advice provided were noted. These findings offer valuable insights into how dietary assessment methods and dietary advice align with wider themes of barriers and facilitators operating across individual (e.g., knowledge and motivation), family (e.g., beliefs and practices) and community levels (e.g., resource availability and policy constraints). Although not interventions themselves, these insights indirectly address domains within the TDF.

Dietary advice provided encompassed five key areas: sugar consumption, infant feeding practices, general dietary advice, snacking habits and body weight management. Dietary advice was delivered through leaflets [32, 39, 45, 52, 57, 60], utilising the waiting room to provide educational information [44], or making referrals to dietitians/nutritionists [39, 45]. Methods for delivering advice included using national dietary guidelines [56], tailoring advice based on dietary assessment [48, 56], talking to caregivers about their observations [45], motivational interviewing [45] and focusing on common risk factors [48, 56]. One study highlighted the importance of discussing food insecurity with families and considering this context when providing dietary advice [55].

Recommendations for infant feeding practices included addressing the types and transition of food and drinks [36], proper bottle or sippy cup use [36, 38, 41, 42, 58], breastfeeding practices [42, 58, 66], and advising against placing sticky/sweet foods on a baby's dummy [60].

Snacking advice emphasised reducing sugary snacks and promoting alternative options like cheese, fruit sticks, yoghurt, chapatis and rice cakes [34, 36, 41, 56, 60, 63]. However, conflicting messages arose from oral health dietary advice leaflets that recommended crisps as a ‘healthy snack [52], conflicting with guidelines which suggest they should be consumed less often and in small amounts [68]. General dietary advice encouraged children to consume fruits and vegetables [32, 56], adopt healthy eating patterns [34, 35, 63], and choose water and milk as drinks [56]. Sugar advice addressed intake, timing, sugar‐sweetened beverage consumption, frequency, amount, sources of hidden sugars and dietary label interpretation [32, 33, 34, 35, 37, 41, 48, 51, 54, 60, 63]. Weight management‐related advice [43] focused on nutritional counselling [31], encompassing general dietary information and behavioural modification programmes [45].

### 3.4 Dietary Assessment

Common dietary assessment methods included verbal questioning [40, 47, 56, 61, 62], diet diaries [41, 46, 47, 48, 49, 54], 3‐day food diaries [61, 62] and 24‐h dietary recalls [35, 47]. One study used a 2‐week food record chart, though adherence was low [54]. Sugar charts and dietetic interns were also used to collect dietary information [35, 56]. When evaluating dietary assessment tools, dental care staff focused on cariogenic/erosive potential of foods, intake volume, frequency and timing, and bedtime consumption [48, 49, 61, 62]. For younger children breastfeeding, bottle‐feeding, age of weaning and feeding patterns were assessed [42]. Some professionals used a common risk factor approach, by identifying dietary behaviours through dietary assessment, to offer tailored advice [48].

## 4 Discussion

This study applied the TDF to identify barriers and facilitators in providing and implementing dietary assessment and/or advice for paediatric populations attending dental clinics. Barriers predominantly aligned with the domains ‘Environmental context and resources’, ‘Knowledge’ and ‘Motivation and goals’. Facilitators, though less frequently reported, corresponded to ‘Social influences’, ‘Skills’ and ‘Motivation and goals’. These findings provide a roadmap for designing future public health interventions to enhance dietary advice and assessment practices in dental settings.

The ‘Environmental context and resources’ domain emerged as a significant barrier, highlighting time constraints and high patient volumes in publicly funded dental clinics. Despite guidelines emphasising the importance of preventive care dental professionals reported insufficient time for meaningful dietary discussions [69]. This issue is particularly detrimental in paediatric dentistry where early dietary guidance is crucial for preventing ECC [70]. Future interventions at midstream dental clinic level, could incorporate brief, standardised dietary assessment tools potentially online into clinical workflows, enabling efficient integration of dietary assessment into routine care. Additionally, inadequate financial incentives for providing dietary advice, particularly in private practice, can discourage dental staff from offering these services [56]. This issue is compounded by evidence showing that some adult patients prioritise paying for physical treatments over preventive care, such as dietary advice, which may further discourage dental care staff from offering these services [71]. Although some adult patients do value preventive advice, it is unclear if these preferences extend to the care they seek for their children. Policies offering financial reimbursement for preventive services could address these structural barriers.

Barriers related to the ‘Knowledge’ domain such as the required knowledge for dietary analysis and unfamiliarity with dietary guidelines discourage some staff from offering dietary advice altogether [42, 56]. A lack of nutritional training in dental education can be a contributing factor, with a survey highlighting only 56% of dentists felt adequately trained in diet and oral health [72]. Upstream public health strategies should emphasise interprofessional education between dietetic and dental students to enhance competency, like one UK‐based institution [73]. Additionally, caregivers can feel overwhelmed by conflicting dietary advice [52, 66], posing a challenge for dental professionals. This confusion may lead caregivers to combine various dietary messages and select information that aligns with their pre‐existing beliefs and personal schemas [38], further complicating effective dietary management. For instance, caregivers may believe that giving children large amounts of pure orange juice, due to its vitamin C content, benefits oral gum health, but its acidic pH can contribute to dental erosion [74]. Therefore, assessing caregivers' baseline knowledge and addressing conflicting information is essential to provide clear, effective dietary advice.

The ‘Motivation and goals’ domain showed that dental care staff often perceived caregivers as unmotivated or uninterested in dietary advice [31, 33, 35, 40, 42, 45, 54]. However, this perception may stem from dental professionals misunderstandings, as caregivers lack of knowledge can be misinterpreted as low motivation and disengagement [54, 75]. This can lead to dental professionals to omit the use of dietary assessment tools or advice, which reduces the chance of identifying at‐risk children. Similarly dental clinic staff's intrinsic motivation, such as personal interest in nutrition emerged as a facilitator. Organisation wide training programmes should utilise behaviour change techniques and leverage intrinsic motivation by highlighting the positive impact dietary advice has on child health outcomes and professional satisfaction [76].

‘Beliefs about capabilities’ domain was prominently reported by caregivers, reflecting their perceived control over their child's dietary behaviours as both a barrier and facilitator. Caregivers reported using cariogenic foods as rewards to manage children's behaviour, a practice recognised by dental care staff [54]. In the UK, children consume more than double the recommended intake of sugar, with 7% coming from sugar confectionary [77]. The use of sweets as rewards, especially those like lollipops that have prolonged exposure in the mouth, contributes significantly to tooth decay and provides no nutritional value [9]. Given the role sugar plays in the development of dental caries, downstream 1–1 interventions should focus on alternative individual behavioural rewards must be considered to mitigate risk. The Theory of Planned Behaviour, helps explain how caregivers with low perceived behavioural control and negative attitudes are less likely to encourage healthy dietary changes in their children oral health behaviour modification [78]. Oral health habits are established early in life and are influenced by parental behaviour, meaning poor dietary practices can increase the risk of ECC, especially if they are a daily occurrence.

The ‘Social/Professional role and identity’ domain highlighted a strong facilitator for dental professionals, emphasising the importance of supporting staff in providing dietary assessment and advice [35, 37, 40, 47, 54]. Interprofessional collaboration in dentistry is linked to improved patient outcomes and greater professional job satisfaction, with dietitians and nutritionists playing an integral role in providing dietary advice related to oral health [35]. By offering personalised dietary advice that considers factors like socio‐economic status, cooking facilities and knowledge, a standard practice of dietitians, they can help address the broader challenges of long‐term dietary behaviour change. However, challenges exist in this interprofessional collaboration, as some dietitians report infrequent referrals in paediatric dental clinics [35]. Dentists have also expressed concern over potential conflicting advice [66], which can limit the effectiveness of preventive efforts. Clarifying professional roles and scope of practice is crucial to maximising patient care success.

This scoping review has some limitations. For example, the cut‐off point for caregivers or dental care staff reporting a barrier was set at 10%. This value was determined in agreement with the research team, based on a previous scoping review using the TDF in the same research field by Lienhart et al. [22]. While 10% may not capture all of the population, it still provides valuable insights into the topic. Given the complexities of providing or receiving dietary advice in the dental settings, this cut‐off allowed for a deeper understanding of the challenges faced by dental professionals and caregivers. Furthermore, the quality of the included studies was not appraised, potentially leading to misinterpretation and risk of bias if poor‐quality studies containing methodological errors were included. Additionally, no analysis of specific sub‐populations within the paediatric population was carried out, limiting its applicability to different public health settings.

## 5 Conclusions

This scoping review highlights the main barriers faced by dental clinic staff when providing dietary assessment/advice to children, which are compounded by the difficulties caregivers encounter in implementing such advice. Environmental factors, such as time constraints and financial reimbursement, were the most common barriers cited by dental professionals. These findings can help researchers and public health policymakers to develop innovative interdisciplinary interventions and systems changes for more effective and personalised dietary care in dental settings.

## Author Contributions

All authors conceptualised the manuscript. L.H. and P.C.‐A. conducted the review. L.H. and P.C.‐A. drafted the manuscript. All authors critically revised the manuscript. All authors gave their final approval and agreed to be accountable for all aspects of the work.

## Conflicts of Interest

The authors declare no conflicts of interest.

## Supporting information


Data S1.

## Data Availability

All data supporting this study is provided as Supporting Information accompanying this paper or provided in full in the results section of this paper.
