# Supplementary material for: Exploring Barriers and Facilitators to Dietary Assessment and Advice in the Paediatric Population Attending Dental Clinics: A Scoping Review
Source: Community Dent Oral Epidemiol. 2025 May 27;53(5):500–13. doi: 10.1111/cdoe.13046 (PMC12423353; doi:10.1111/cdoe.13046)
Supplement: Supplementary file 1 — Data S1. [file CDOE-53-500-s001.docx]

**Supplementary material**

Supplementary table 1. Boolean search strategy on four databases

| Database | Search strategy |
| --- | --- |
| MEDLINE (EBSCO) | \| 18 \| S4 AND S10 AND S16 \| \| --- \| --- \| \| S17 \| S4 AND S10 AND S16 \| \| S16 \| S11 OR S12 OR S13 OR S14 OR S15 \| \| S15 \| AB ( child* OR preschool OR adolescent* OR infant* OR paediatric* OR pediatric* ) OR TI ( child* OR preschool OR adolescent* OR infant* OR paediatric* OR pediatric*) \| \| S14 \| (MH "Infant") \| \| S13 \| (MH "Adolescent") \| \| S12 \| (MH "Child, Preschool") \| \| S11 \| (MH "Child") \| \| S10 \| S5 OR S6 OR S7 OR S8 OR S9 \| \| S9 \| AB ( "dental setting*" OR "dentistry setting*" OR "dental visit*" OR "dental clinic*" OR "dental office*" OR dentist* ) OR TI ( "dental setting*" OR "dentistry setting*" OR "dental visit*" OR "dental clinic*" OR "dental office*" OR dentist* ) \| \| S8 \| (MH "Dentistry") \| \| S7 \| (MH "Dentists+") \| \| S6 \| (MH "Dental Offices") \| \| S5 \| (MH "Dental Clinics") \| \| S4 \| S1 OR S2 OR S3 \| \| S3 \| AB ( diet* OR food OR nutrition* ) OR TI ( diet* OR food OR nutrition* ) \| \| S2 \| (MH "Food+") \| \| S1 \| (MH "Diet+") \| |
| Cochrane Database of Systematic Reviews (Wiley) | #1 MeSH descriptor: [Diet] explode all trees  #2 MeSH descriptor: [Food] explode all trees  #3 diet* OR food OR nutrition*  #4 #1 OR #2 OR #3  #5 MeSH descriptor: [Dental Clinics] explode all trees  #6 MeSH descriptor: [Dentists] explode all trees  #7 MeSH descriptor: [Dentistry] this term only  #8 (dental NEXT setting*) OR (dentistry NEXT setting*) OR (dental NEXT visit*)  OR (dental NEXT clinic*) OR (dental NEXT office*) OR dentist*  #9 #5 OR #6 OR # 7 OR #8  #10 MeSH descriptor: [Child] explode all trees  #11 child* OR preschool OR adolescent* OR infant* OR paediatric* OR pediatric*  #12 #10 OR #11  #13 #4 AND #9 AND #12 |
| Embase (ovid) | \| 1 . \| exp diet/ \| \| --- \| --- \| \| 2. \| exp food/ \| \| 3. \| nutrition/ \| \| 4. \| (diet* or food or nutrition*).ti. or (diet* or food or nutrition*).ab. \| \| 5. \| 1 or 2 or 3 or 4 \| \| 6. \| exp dental clinic/ \| \| 7. \| exp dentist/ \| \| 8. \| dentistry/ \| \| 9. \| ("dental setting*" or "dentistry setting*" or "dental visit*" or "dental clinic*" or  "dental office*" or dentist*).ti. or (“dental setting*” or “dentistry setting*”  or “dental visit*” or “dental clinic*”or “dental office*” or dentist*).ab. \| \| 10. \| 6 or 7 or 8 or 9 \| \| 11. \| exp child/ \| \| 12. \| preschool child/ \| \| 13. \| adolescent/ \| \| 14. \| infant/ \| \| 15. \| exp pediatrics/ \| \| 16. \| (child* or preschool or adolescent* or infant* or paediatric* or pediatric*).ti. or  (child* or preschool or adolescent* or infant* or paediatric* or pediatric*).ab. \| \| 17. \| 11 or 12 or 13 or 14 or 15 or 16 \| \| 18. \| 5 and 10 and 17 \| |
| CINAHL (EBSCO) | \|  \| \| --- \| \| S17 \| S4 AND S10 AND S15 \| \|  \| \|  \| S16 \| S4 AND S10 AND S15 \| \| \|  \| S15 \| S11 OR S12 OR S13 OR S14 \| \| \|  \| S14 \| TI ( child* OR preschool OR adolescent* OR infant* OR paediatric* OR  pediatric* ) OR AB ( child* OR preschool OR adolescent* OR infant* OR  paediatric* OR pediatric* ) \| \| \|  \| S13 \| (MH "Adolescence") \| \| \|  \| S12 \| (MH "Infant") \| \| \|  \| S11 \| (MH "Child") \| \| \|  \| S10 \| S5 OR S6 OR S7 OR S8 OR S9 \| \| \|  \| S9 \| TI ( "dental setting*" OR "dentistry setting*" OR "dental visit*" OR  "dental clinic*" OR "dental office*" OR dentist* ) OR AB  ( "dental setting*" OR "dentistry setting*" OR "dental visit*" OR  "dental clinic*" OR "dental office*" OR dentist* ) \| \| \|  \| S8 \| (MH "Dentistry") \| \| \|  \| S7 \| (MH "Dental Offices") \| \| \|  \| S6 \| (MH "Dental Clinics") \| \| \|  \| S5 \| (MH "Dentists") \| \| \|  \| S4 \| S1 OR S2 OR S3 \| \| \|  \| S3 \| TI ( diet* OR food OR nutrition* ) OR AB ( diet* OR food OR nutrition* ) \| \| \|  \| S2 \| (MH "Diet+") \| \| \|  \| S1 \| (MH "Nutrition+") \| \| |

Supplementary table 2. Characteristics of studies included in the scoping review

| **Author reference, country** | **Study aims** | **Study design**  **(population)** | **Person providing dietary assessment/advice** | **Dietary assessment provided** | **Dietary advice provided** | **Setting where advice is given/ dentists practice sector** |
| --- | --- | --- | --- | --- | --- | --- |
| Albadri, Allen and Ajeigbe, 2024, ^55^ UK | To explore current awareness and understanding of food insecurity within the dental team. It examines the levels of confidence and understanding amongst the team regarding the importance of general dental health education. | Questionnaire (n=76) | Dental professionals, working with children | X | ✓ | Hospital dental service, community dental service and general dental practice. |
| Arheiam *et al*. 2016 ^47^, UK | To investigate the prevalence and frequency of diet diary use in English dental practices and to examine the factors which influence their use. | Questionnaire  (n=250) | General dental practitioner | ✓ | X | NHS and private sector |
| Arheiam et al. 2018 ^46^, UK | To explore the reasons for poor adherence to diet-diaries issued to children in a dental hospital setting. | Qualitative collective case study: observation of dentist- patient interactions (n=11), interviews with the child/caregiver dyads and dentists (n= 14) and documentary analysis of returned diet diaries | Dentists | ✓ | X | Hospital setting |
| Arheiam et al. 2016,^48^ UK | To explore how dentists integrate information from diet diaries to deliver usable advice to patients. | Questionnaire  (n=229) | General dental practitioners | ✓ | ✓ | NHS and private sector |
| Arheiam et al. 2016 ^49^, UK | To investigate associated factors affecting diet diaries return rate and the information obtained from returned diaries. | Retrospective study  (n=174) | Does not specify the professional that issued the dietary diary. | ✓ | X | Dental hospital |
| Arora et al. 2012 ^60^, Australia | To record how caregivers cope with dental health education materials for preschool children commonly available in New South Wales, Australia and to identify barriers which prevent them from practicing the advice given. | Semi -structured interviews  (n=24) | Dental leaflets | X | ✓ | NI |
| Arora et al. 2022 ^56^ , Australia | To explore the perceptions of oral health care professionals on childhood overweight and obesity screening and management in oral health settings in the Greater Sydney region in New South Wales. | Semi- structured interviews  (n=15) | Dental therapists/ oral health therapists, general dentists and paediatric dentists | ✓ | ✓ | Public oral health services and a combination of public and private practice. |
| Braithwaite et al. 2008 ^31^, USA | To document current nutrition/healthy lifestyle practices of paediatric dentists in North Carolina and examine factors associated with those practices. | Survey  (N= 70) | Paediatric dentists | X | ✓ | Private practice, public health settings and hospital settings |
| Cashmore et al. 2011 ^57^, Australia | To explore participating caregivers’ experiences (of the Tooth Smart Program) of and views about caregiver counselling; identify and describe factors that influence uptake of oral health advice; and uncover any anticipated outcomes of caregiver counselling | Semi-structured interviews  (n=14) | Dental therapists and dental assistants | X | ✓ | Hospital dental setting |
| Chang et al. 2018 ^32^, USA | To explore how content and format of children’s oral health instruction in the dental clinic is perceived by caregivers and might affect caregivers’ knowledge and behaviours. | Interviews  (n= 30) | Dentists | X | ✓ | Local dental clinic |
| Clovis et al. 2012 ^33^, USA | To assess Maryland dental hygienists’ knowledge, practices and opinions regarding dental caries prevention and early diagnosis. | Survey  (n=540) | Dental hygienists | ✓ | ✓ | Solo practice and group settings.  A combination of private insurance, out of pocket and Medicaid. |
| Cole et al. 2018 ^34^,USA | To explore dental hygienists’ beliefs, attitudes, knowledge, current practices and barriers for assessing and educating patients about childhood obesity. | Survey  (n=919) | Dental hygienists | X | ✓ | General practice, paediatric practice and other |
| Fernandez et al. 2017 ^35^, USA | To explore how experiences in the paediatric dental clinic have influenced beliefs and clinical practice regarding children’s oral health and nutrition among registered dietitians who completed the elective oral rotations during their internships. | Questionnaire  (n=36) | Dietitians | ✓ | ✓ | New York University paediatric dentistry clinic. |
| Gussy et al. 2006 ^58^, Australia | To explore the oral health beliefs and practices of primary health care professionals which may act as barriers to the development of a model of shared care for the oral health of pre-school children. | Semi-structured interviews and focus groups  (n=56) | Maternal and child health nurses, paediatricians, dental professionals (dentists, dental nurses, and dental practice managers) and general medical practitioners. | X | ✓ | Variety of medical settings |
| Hambire and Hambire 2022 ^61^, India | To explore the perceptions of the paediatric dentists regarding the possible difficulties and their solutions for the usage of diet diaries in their dental office. | Questionnaire  (n= 314)  And semi-structured interviews and observing the paediatric patients and their caregivers. | Paediatric dentists | ✓ | ✓ | Dental hospital |
| Hambire et al. 2022 ^62^, India | To explore the perceptions of the paediatric dentists regarding the possible difficulties and their solutions for the usage of diet diaries in their dental office. | Questionnaire  (n= 314)  And semi-structured interviews and observing the paediatric patients and their caregivers. | Paediatric dentists | ✓ | ✓ | Dental hospital |
| Henderson et al. 2015 ^50^, UK | To assess the acceptability to caregivers, dental practice staff and commissioners of the delivery of dietary advice in the dentistry setting in order to address obesity. | Semi structured focus groups with dental practice staff (n=23) and interviews with caregivers (n=4) and commissioner (n=1). | Dental receptionists, dental assistants, dental nurses, dental hygienists, dentists and practice managers | X | Explored dentists’ perceptions on providing dietary advice related to obesity. | Primary care |
| Hoeft et al. 2015 ^36^, USA | To describe qualitative findings of the initial acceptability of curriculum content and activities, present the process of refinement of the curriculum through engaging the target population and *promotoras* and presents results from the evaluation assessing the acceptability of the curriculum once implemented. | Focus group (n=51)  Surveys based on *Contra Caries* Oral Health Education Program  (n=83) | Promotoras – hired and trained caregivers to deliver oral health advice through *contra caries* sessions | X | ✓ | - |
| Holloway et al. 1994 ^51^, UK | To explore views of established, successful general dental practitioners treating their child patients under a capitation system of renumeration, in order to discover what preventive procedures on which patients they considered were of benefit to their practices and why. | Quantitative interviews (n=50) and discussion groups (n=21) | General dental practitioners | X | ✓ | Not specified (but under capitation) |
| Horowitz et al. 2017 ^37^, USA | To gain an in-depth understanding of dental hygienists and dentists’ perspectives regarding children’s oral health and what needs to be done to prevent early childhood caries. | Focus groups and semi- structured interviews  (n=37) | Dental hygienists, general and paediatric dentists | ✓ | ✓ | Not specified (but participants must accept Medicaid patients). |
| Horton et al. 2008 ^38^, USA | To examine Latino immigrant caregivers’ explanatory models of the causes of early childhood caries. | Interviews  (n= 38) | Educators at the federal Women, Infants and Children (WIC) nutritional program or dentists | X | ✓ | X |
| Huang et al. 2006 ^39^, USA | To determine clinician opinions regarding the effect of childhood overweight on medical and dental health and to assess the current status of overweight screening practices and nutrition education offered to paediatric patients and their families at orthodontic offices. | Survey  (n=111) | Orthodontists | ✓ | ✓ | Patients who self-pay, insured or those receiving Medicaid |
| Moon et al. 1998 ^65^, Korea | To determine the level of knowledge and opinions about caries aetiology and prevention among Korean dentists and to describe related factors | Questionnaire  (n=1700) | General dental practitioners | X | ✓ | Private practice and public health centre |
| Morgan et al. 2010 ^52^, UK | To determine whether oral health education leaflets with a food and nutritional focus conform to existing UK national nutritional guidelines. | Oral health education leaflets (n=30) | X | X | ✓ | Leaflets were selected from UK community dental clinics, dental hospitals and training and education centre for dental care professionals. |
| Naidu et al. 2012 ^66^, Trinidad | To explore and understand caregivers and caregivers experience of oral healthcare for their preschool aged children and how, within their own social context, this may have shaped their oral health attitudes and behaviours. | Focus group  (n=18) | Dentists and doctors | X | ✓ | Caregivers attended both private and public dental care facilities |
| Nandi et al. 2023 ^40^, USA | To explore how interprofessional education with dietetic interns in training has influenced views and practices of paediatric dentists after graduation. | Survey  (n=44) | Paediatric dentists | ✓ | ✓ | Private practice, academia/research, hospital setting and public health/community-based settings. |
| Parry et al. 2023 ^67^, Does not specify location. | To examine parental perceptions of difficulties associated with dental attendance and oral care for autistic children and young adults to highlight reported challenges and potential adaptations, and to identify interventions that will encourage positive experiences of dental attendance. | 2 focus group  (n=10) | X | X | ✓ | Not specified |
| Sajnani-Oommen et al. 2006 ^41^, USA | To compare the provision of attitudes toward nutritional counselling between paediatricians and paediatric dentists. | Questionnaire  (n=325) | Paediatric dentists and paediatricians | ✓ | ✓ | Private practice and hospital/academic affiliations. |
| Schofield et al. 2022 ^53^, UK | To determine the knowledge of nursing teams within a children’s hospice and the carers of those children and young people who use the service. Through carrying out a quality improvement project to develop mouth care, the aim was to investigate whether the mini mouthcare matters programme should be recommended for a wider implementation in children’s hospices. | Questionnaire  (n=67) | Nursing colleagues, healthcare assistants and physiotherapists | X | ✓ | Hospice setting |
| Shqaidef 2021 ^64^, Jordan | To investigate the comprehension of consent among the caregivers of orthodontic patients undergoing fixed orthodontic treatment using verbal explanation supported with the University Hospital consent leaflet. | Questionnaire  (n=32) | Orthodontist written and verbal consent form | X | ✓ | University Hospital |
| Sim et al. 2013 ^42^, USA | To describe practice patterns, knowledge and attitudes of paediatric dentists in North Carolina in delivering dietary recommendations to the caregivers/caregivers of infants and toddlers; and identify barriers that limit the implementation of related recommendations. | Survey  (n=86) | Paediatric dentists | ✓ | ✓ | Private practice and public health/community clinic |
| Smith et al. 2021 ^43^, USA | To assess dental students’ attitudes, comfort and perceived barriers discussing nutrition and obesity prevention with caregivers and caregivers of children aged 0-5, after a one-time service-learning experience in a paediatric primary care setting to promote oral health. | Survey  (n=101) | Second year dental students | X | ✓ | University paediatric outpatient clinic |
| Tamura et al. 2020 ^63^, Japan | To clarify the importance of dental support for eating problems of infants, young children and disabled children, based on a questionnaire survey administered to dentists and guardians. | Questionnaire (n= 712 dentists and 844 guardians) | Dentists treating paediatric patients | X | ✓ | X |
| Threlfall et al. 2007 ^54^, UK | To increase understanding about how and to whom general dental practitioners provide preventive advice to reduce caries in young children. | Semi-structured interviews  (n=93) | General dental practitioner | ✓ | ✓ | NI |
| Tiwari et al. 2017 ^44^, USA | To describe oral health knowledge, behaviours and beliefs of Latino caregivers with children under the ages of 6 years and to conduct a needs assessment with Latino families to better understand the challenges in maintaining oral health for their children. | Focus groups  (n=30) | Informative video. | X | ✓ | Dental clinics |
| Villarosa et al. 2022 ^59^, Australia | To codesign guideline implementation strategies for children’s growth assessment and dietary advice guidelines in the dental setting. | Focus groups  (n=20) | Dental therapists, oral health therapists and dental assistants. | X | ✓ | Public dental clinics |
| Wright and Casamassimo 2017 ^45^, USA | To determine attitudes, behaviours, future intentions and perceived barriers of paediatric dentists regarding efforts to prevent childhood obesity and reduce children’s consumption of sugar sweetened beverages. | Survey  (N=1615) | Paediatric dentists | X | ✓ | Private practice, academia/research, public health/community clinic, hospital-based clinic, government and corporate. |

✓ indicates dietary assessment provided or dietary assessment provided was described in the paper.

X represents dietary assessment provided or dietary assessment provided was not indicated in the paper.

Supplementary table 3. Frequency of reported barriers and facilitators for each TDF domain

| TDF Domain | Number of times identified as a barrier to  Dental Clinic Staff | Number of times identified as a facilitator to  Dental Clinic Staff | Number of times identified as a barrier to  Caregivers | Number of times identified as a facilitator to  Caregivers |
| --- | --- | --- | --- | --- |
| **Knowledge** | 15 | 4 | 6 | 2 |
| **Skills** | 8 | 13 | 0 | 0 |
| **Social/Professional role and identity** | 5 | 14 | 3 | 0 |
| **Beliefs about capabilities** | 4 | 0 | 8 | 2 |
| **Beliefs about consequences** | 12 | 8 | 6 | 0 |
| **Motivation and goals** | 19 | 10 | 1 | 0 |
| **Memory, attention and decision processes** | 0 | 5 | 2 | 0 |
| **Environmental context and resources** | 40 | 5 | 4 | 3 |
| **Social influences** | 5 | 0 | 7 | 1 |
| **Emotion** | 5 | 0 | 9 | 3 |
| **Behavioural regulation** | 4 | 1 | 3 | 1 |
| **Nature of the behaviours** | 5 | 0 | 1 | 1 |

Supplementary information 1. Additional Journals manually searched

1. International Journal of Paediatric Dentistry
2. Paediatric Dentistry
3. Community Dentistry and Oral Epidemiology,
4. British Dental Journal
5. Journal of Public Health Dentistry
6. International Dental Journal
7. BMC Oral Health
8. Journal of the American Dental Association
9. European Journal of Paediatric Dentistry
